# Evidence-based decision making for malaria elimination applying the Freedom From Infection statistical framework in five malaria eliminating countries: an observational study

**DOI:** 10.1016/S2214-109X(25)00236-0

**Published:** 2025-08-19

**Authors:** Gillian Stresman, Luca Nelli, Lindsey Wu, Isabel Byrne, Henry Surendra, Bryan Fernandez-Camacho, Jorge Ruiz-Cabrejos, Lucia Bartolini Arana, Adéritow Augusto Lopes Macedo Gonçalves, Davidson Daniel Sousa Rocha Monteiro, Luccene Desir, Keyla Ureña, Manuel de Jesus Tejada Beato, Elin Dumont, Monica Hill, Lynn Grignard, Sabrina Elechosa, Raymart Bunagan, Nguyen Xuan Thang, Nguyen Thi Huong Binh, Nguyen Thi Hong Ngoc, Kevin K A Tetteh, Gregory S Noland, Karen E S Hamre, Silvânia da Veiga Leal, Adilson DePina, Ngo Thang, Fe Esperanza Espino, Gabriel Carrasco-Escobar, Jason Matthiopoulos, Chris Drakeley

**Affiliations:** a Department of Epidemiology, College of Public Health, University of South Florida, Tampa, FL, USA; b Department of Infection Biology, London School of Hygiene & Tropical Medicine, London, UK; c School of Biodiversity, One Health and Veterinary Medicine, University of Glasgow, Glasgow, UK; d Monash University Indonesia, Tangerang, Indonesia; e Oxford University Clinical Research Unit Indonesia, Faculty of Medicine, Universitas Indonesia, Jakarta, Indonesia; f Health Innovation Laboratory, Institute of Tropical Medicine, Universidad Peruana Cayetano Heredia, San Martín de Porres, Peru; g Laboratório de Entomologia Médica, Instituto Nacional de Saúde Pública, Praia, Cabo Verde; h Hispaniola Initiative, The Carter Center, Atlanta, GA, USA; i Centro de Prevención y Control de Enfermedades Transmitidas por Vectores y Zoonosis, Ministerio de Salud Pública, Av Dr Héctor Homero Hérnandez, Esq Av Tiradentes, Ens La Fe Santo Domingo, Dominican Republic; j Section of Virology, School of Biosciences, University of Surrey, Guildford, UK; k Department of Parasitology, Research Institute for Tropical Medicine, Alabang, Muntinlupa, Metro Manila, Philippines; l Molecular Biology Department, Vietnam National Institute of Malariology, Parasitology and Entomology, Ha Noi, Viet Nam; m Global Health and Tropical Medicine, Instituto de Higiene e Medicina Tropical, Universidade Nova de Lisboa, Lisbon, Portugal; n National Malaria Elimination Program, CCS-SIDA, Praia, Cabo Verde

## Abstract

**Background:**

Routine surveillance is a pillar of malaria programmes, and the primary source of data used for decision making. However, any inference when relying on routine data to inform decision making is limited by how effective the system is at measuring the actual malaria burden. Here, we aimed to extend the Freedom From Infection (FFI) framework to produce species-specific estimates of surveillance system sensitivity and probability of freedom from malaria, combine multiple surveillance components including community case management and active case detection, and apply the FFI model in five malaria eliminating settings.

**Methods:**

Monthly routine data on *Plasmodium falciparum* and *Plasmodium vivax* and health system factors were collected from 1515 facilities across five countries. Additionally, data from 12 community health workers and from 10 767 individuals from cross-sectional surveys (active case detection) were available. The data were analysed using FFI models accounting for multiple malaria species and surveillance components. The primary outcomes were the sensitivity of the surveillance system and the probability of malaria freedom.

**Findings:**

Strong surveillance systems were characterised by access to testing and treatment supplies, training on diagnostics and case management within the previous 12 months, and shorter estimated travel times to facilities. Only half of the facilities (841 of 1515 facilities for *P falciparum* and 771 of 1455 facilities for *P vivax*) had sufficient sensitivity to achieve and maintain a high probability of freedom, consistent with having achieved malaria elimination, with either passive case detection data alone or when combined with active case detection.

**Interpretation:**

Applying the FFI model framework to malaria surveillance data can provide programmes with information to support decision making, specific to malaria species. When routine malaria surveillance systems are strong, they are sufficient to achieve and maintain a high probability of freedom. Including additional surveillance components such as community case management and active case detection with multiple diagnostic tools can help improve estimates for which routine malaria data alone are not sufficient to ensure confidence in elimination.

**Funding:**

The Bill and Melinda Gates Foundation, the Global Institute for Disease Elimination, and the Carter Center.

## Introduction

Data-driven decision making is a cornerstone of public health and surveillance is a core pillar of the Malaria Global Technical Strategy, 2016–2030.^1^ Malaria passive surveillance systems provide an important source of information with which to monitor disease trends, measure the impact of interventions, and ensure malaria programmes are adapted accordingly.^2^ However, any inference using routine malaria data is limited by how effective the system is at measuring the burden. When the goal of a surveillance system is to monitor disease trends, counting cases is usually sufficient to inform decisions, even if the system is imperfect.^3^ When the goal is to strengthen the surveillance system or provide evidence to support claims of elimination, a quantitative approach that accounts for any uncertainty in the data could provide an important tool to support programmatic decision making.

The global burden of malaria is high, with an estimated 263 million malaria cases in 2023, of which 3·5% were due to *Plasmodium vivax*.^4^ Maintaining a robust malaria surveillance system is crucial for generating evidence to support claims of elimination. Applying a tool that can assess the strength of the surveillance system has the distinct advantage that the claims of elimination can be data-driven and account for uncertainty. Crucially, a quantitative approach that can account for uncertainty in the resulting estimates; identify specific facets of a strong health system and consequently factors that could be targeted for improvement; and estimate the likelihood that elimination has been achieved, would add value to the largely qualitative approaches in use.^1^, ^5^

**Research in context**

**Evidence before this study**

A strong surveillance system is a crucial foundation in the global strategy for malaria control and elimination. Despite recent progress (ie, within the past 5–10 years) in strengthening data quality and cultures of data use, the tools available to assess the reliability of malaria surveillance systems are largely composed of qualitative methods or descriptive analysis, while the quantitative measures used are typically measures of efficiency being used as a proxy for how effective the system is at case detection. Previous work has adapted a method developed from veterinary epidemiology for use with human malaria, providing estimates of the sensitivity of the surveillance system and the probability of freedom from malaria (ie, the likelihood that elimination is consistent with the data available). However, malaria transmission and the associated surveillance components being implemented in different settings are complex and multifaceted. For the Freedom From Infection (FFI) approach to complement the existing surveillance landscape and be useful as a programmatic tool for the context of malaria, ensuring that both surveillance system and probability of freedom estimates are specific to all species of Plasmodium parasites and can account for different types of surveillance data being collected in elimination settings is not currently possible. In January, 2023, PubMed (n=155) and Scopus (n=2574) databases were searched for articles in English or French published between January, 2007, and December, 2022, on malaria surveillance for elimination and data quality. Only four papers were identified that described or applied quantitative approaches for estimating the sensitivity of the surveillance system and/or the probability that malaria elimination is likely based on the available malaria surveillance data.

**Added value of this study**

Our work has adapted the initial FFI model, which focused on incorporating routine malaria surveillance data only and was agnostic to malaria species. Key developments presented include generating species-specific outputs for *Plasmodium falciparum* and *Plasmodium vivax* and incorporating data generated through community case management and active case detection. Additionally, the models were adapted to account for the use of different malaria diagnostic tools, including metrics of infection and exposure, in household surveys. To test the updated model framework, we collected data from 1515 health facilities in five malaria eliminating countries. We found that in these settings, where the surveillance system sensitivity is good, routine malaria passive case detection surveillance data alone are sufficient to obtain a high probability of freedom and provide confidence that elimination has been achieved. Additionally, we identified several factors associated with a strong surveillance system that are largely consistent across the included settings on three continents. When the passive case detection data alone were insufficient to achieve a high certainty in surveillance system sensitivity or probability of freedom, the addition of data from community case management and active case detection improved estimates: of the 10 767 people included in the surveys, there were 0 infections detected by rapid diagnostic test, microscopy, or molecular methods for either species.

**Implications of all the available evidence**

To the best of our knowledge, our results are the first to apply the extended FFI model framework to estimate the surveillance system sensitivity and probability of malaria freedom per health facility from five malaria eliminating settings across three continents. Our evidence suggests that despite differences in surveillance systems and malaria epidemiology, strong surveillance systems share common characteristics. The FFI model provides a quantitative framework that programmes can use to inform interventions designed to strengthen the surveillance system or to provide a baseline to assess the effect of any changes. Similarly, when the goal is to eliminate malaria transmission, the probability of malaria freedom metric could provide a benchmark for programmes while simultaneously identifying areas in which additional efforts might be needed. Notably, we have shown that a strong surveillance system is sufficient to have confidence in malaria elimination, but this framework provides additional information that can identify and target where additional efforts to strengthen or supplement malaria surveillance might be of most benefit. Ultimately, the FFI framework provides a tool that can provide an important quantitative framework to complement existing malaria data and surveillance assessment guidelines.

The Freedom From Infection (FFI) framework, described by Nelli and colleagues,^6^, ^7^ provides a probabilistic approach for evaluating malaria surveillance systems and is particularly relevant to the context of malaria elimination. Briefly, this method uses a hierarchical Bayesian model to estimate two critical metrics: the sensitivity of the surveillance system and the probability of malaria freedom. These estimates integrate data from routine malaria surveillance, such as passive case detection, with supplementary components such as community case management and active case detection. By modelling both observed data and latent malaria transmission dynamics, the framework explicitly accounts for passive case detection data being an incomplete representation of malaria transmission, as well as uncertainties in data quality, health system performance, expected health system engagement, and spatiotemporal malaria patterns.^6^

For such a framework to support programmatic decision making, the model should be broadly applicable across surveillance systems and malaria transmission landscapes, while applying a consistent standard for what constitutes elimination. Therefore, the main objective of this research was to determine whether the FFI framework could be consistently applied to the context of malaria in different settings and to generate evidence for the use of a tool to support programmatic decision making in the context of malaria elimination. Here, we present results showing how the initial FFI framework has been extended to account for different contexts in five malaria eliminating countries: Cabo Verde, Dominican Republic, Peru, the Philippines, and Viet Nam. In this observational study, we used data from routine malaria surveillance systems, did key informant interviews to assess health system capacity for malaria, and implemented cross-sectional surveys. Within each setting, we aimed to estimate the sensitivity of the surveillance system, identify factors that promote a strong health system for detecting malaria, and calculate the corresponding probability of malaria freedom. We show the impact of including different surveillance components, comprising active case detection and community case management, present the results of extending the model to produce species-specific estimates, and show application of this tool at scale.

## Methods

### Data collection

The areas within the five study countries consistently reporting zero cases were purposely selected to represent a range of malaria transmission and health systems to test different facets of the FFI framework (appendix p 2).

At each of the facilities within the identified study areas where no locally acquired malaria cases were reported for at least the previous 2 years, monthly routine malaria surveillance data were collected (appendix p 5). When routine malaria data were available in electronic format at the facility-month scale (ie, in Cabo Verde and the Philippines), they were obtained from the national malaria programme. In the other settings, the monthly aggregated data were collected directly from paper records at participating health facilities. When available, the data were recorded separately for *Plasmodium falciparum* and *P vivax.* Passive case detection data time-periods were collected according to record availability and ranged from 3 years to 13 years (appendix p 2).

To estimate the probabilities in the care-seeking cascade, key informant interviews were conducted to collect information on health system factors.^7^ Staff involved with malaria diagnosis and treatment in the included facilities were purposively selected. Information collected included testing and treatment practices, availability of trained staff and testing equipment, stockouts of key supplies (eg, diagnostic tests), the size of the catchment population, and any trainings on case management or testing practices within the previous 12 months. Additional questions focused on quality assurance processes for malaria diagnosis as well as practices around the reporting of malaria surveillance data. Interviews were conducted in person or by phone. In Cabo Verde, the survey was administered using an online form to reach all health facilities across nine islands. In the Dominican Republic, the same procedures were used to collect routine data and conduct interviews with the community health workers, using modified forms relevant for the role. The community health workers in the Dominican Republic are expected to screen their assigned populations for symptomatic malaria monthly by rapid diagnostic test. Finally, the average travel time to each facility was calculated based on derived catchment areas as a proxy for access to health facilities to account for the established relationship between people residing further from a health facility being less likely to seek care for malaria.^6^, ^7^, ^8^, ^9^, ^10^

Observational surveys were designed in three of the five countries to test different use-cases where active case detection could supplement passive case detection. Health facility catchment area was the primary sampling unit, with the sampling strategy designed to address the facets of the malaria risk unique to each setting that would augment corresponding probability of freedom estimates.

In Cabo Verde, the threat to maintaining malaria elimination is imported infections associated with travel from endemic African countries.^11^ We used respondent-driven sampling that targeted adults (ie, those aged >20 years) who had travelled to the African continent in the previous 4 weeks.^12^, ^13^, ^14^ To account for the risk of onward transmission associated with imported infections, we included a reactive component targeting index cases defined as rapid diagnostic test positives and a simple 25% random selection of individuals with negative rapid diagnostic tests. All consenting residents in index case households and five immediate neighbouring households were targeted for inclusion. In Peru, access to care is a known challenge and thus, people who reside further from a health facility are expected to be less well represented by routine surveillance data.^15^ Communities were selected stratified by distance to the nearest health facility to address this. Next, in Viet Nam, the large number of health facilities included in the study enabled us to assess the feasibility of this tool at scale and how different approaches for powering the probability of freedom boost at the facility level and district level could be incorporated, using simple and two-stage random sampling strategies.

The study procedures were the same across all three countries. All individuals residing in selected households older than 6 months (3 months of age in Peru as based on local norms for malaria surveys) before the survey were eligible for inclusion. After obtaining informed consent, a questionnaire was distributed to collect information on demographics, use of vector control, care-seeking behaviours, and malaria history. A fingerpick blood sample was collected to test for malaria infection by rapid diagnostic test (in Cabo Verde and Viet Nam) and microscopy (in Peru and Viet Nam) and to collect spots on Whatman 3 mm filter paper. The decision on whether rapid diagnostic test and/or microscopy was used as a point-of-care test was informed by national guidelines (appendix p 2). The filter paper sample was used to test for *P falciparum* and *P vivax* infection using PCR and recent (ie, exposure in the past 12 months) and historical exposure for both species using Luminex (appendix p 7).^16^, ^17^, ^18^

Permission to collect the routine malaria data was obtained by the ministries of health in each country and written informed consent to conduct the health system interview was obtained from each participant. For participation in the active case detection, individual written informed consent was obtained. For those under the age of majority (ie, those aged 18 years), consent was provided by the primary caregiver, with older children (ie, those aged 7 years to 17 years) also providing assent. The study protocols were approved by: Conselho Nacional De Ética para a Investigaçao, Cabo Verde 80/2020; Consejo Nacional de Bioética en Salud, Dominican Republic 1448; Emory STUDY00003770; Universidad Peruana Cayetano Heredia, Peru 201615; the Research Institute of Tropical Medicine, Philippines 2020–31; the Viet Nam National Institute of Malariology, Parasitology, and Entomology 41/QD-VSR; and the London School of Hygiene & Tropical Medicine 17927/21886/26600/19167/21697.

### Data analysis—applying the FFI framework

The FFI model framework has been described in detail by Nelli and colleagues.^6^ Briefly, the statistical framework models the potential unobserved (latent) dynamics of malaria transmission in the population and the observed malaria cases in the health system simultaneously as part of a joint Bayesian state-space framework, accounting for similarities in the dynamics of neighbouring regions. The model first estimates the probabilities associated with each step in the care-seeking cascade for an infected individual to be detected via passive case detection. These estimates are applied to the latent component of the model to determine any unobserved malaria trends that could be present in the community based on available malaria data and surveillance system capacity. When the observed trends in the health facility are consistent with the expected trends in the community, a facility is considered to have a strong surveillance system, and when fewer than one case per 10 000 population are reported, this translates into a high probability of elimination, here defined as achieving a 95% probability of freedom or greater.

Next, the FFI model framework was extended to account for scenarios commonly encountered within malaria surveillance. With the data available in the five settings, model extensions were applied to show the impact of incorporating multiple surveillance components including community case management and active case detection, and multiple malaria diagnostic tests including both measures of infection and markers of recent and historical malaria exposure (appendix p 8). Similarly, the results of generating species-specific estimates of the surveillance system and the probability of freedom and feasibility at scale were shown. Posterior predictive checks were conducted to validate and assess model accuracy.

### Role of the funding source

The funders of the study had no role in study design, data collection, data analysis, data interpretation, or writing of the report. The Carter Center provides salary support for LD, GSN, and KESH.

## Results

This study included data from 1515 facilities in five countries representing 69·8 million outpatient visits and 1·16 million malaria tests over 446 observation months (country range 52–156). Passive case detection across all five study areas reported 40 056 cases of *P falciparum* and where this species was or is endemic, 167 693 *P vivax* cases were also reported. In the Dominican Republic, community health workers tested 29 554 people over 36 months and identified 736 cases of *P falciparum*. Zero malaria infections by PCR were detected in the 10 767 people sampled as part of active case detection (appendix p 13). Low levels of recent and historical exposure to malaria were observed in all study populations, with trends consistent with the known epidemiology (appendix p 13).

**Figure 1:**
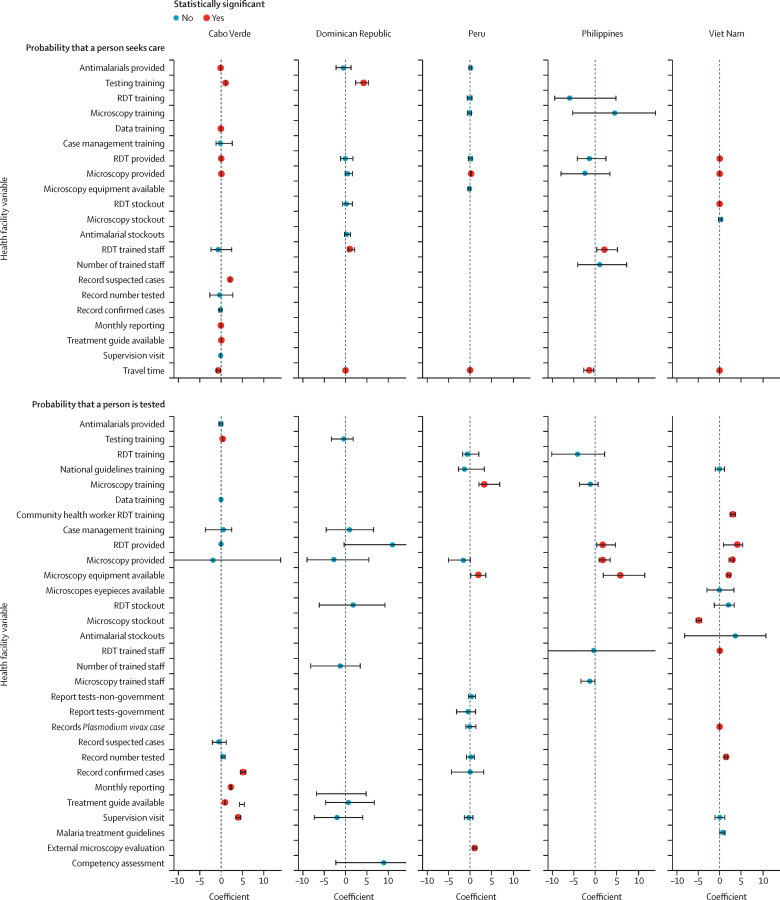
Results of models to estimate the probability of seeking care and the probability that a person is tested for malaria Results of the models per country are shown in the columns. The specific variables included in the models are listed on the y axis with the corresponding coefficient value on the x axis. The estimated coefficient value (point) and associated 95% credible interval (black horizontal lines) are shown per variable. Red and blue coloured points represent the variables that were and were not statistically significant (p<0·05), respectively. The extreme values of select credible intervals for some values for the probability that a person is tested for malaria were cut off and included as text annotation for visualisation purposes. RDT=rapid diagnostic test.

The probability of seeking care was positively associated with facilities having testing and treatment supplies and trained staff (figure 1). People were less likely to seek care when there were stockouts of testing or treatment supplies and with increased average travel time to visit a facility. People were more likely to be tested for malaria in facilities that had quality assurance processes, testing equipment, and trained staff, and in those consistently reporting their malaria surveillance data (figure 1). People were less likely to be tested where there were stockouts of testing equipment. The other steps in the care-seeking cascade, including the probability that a person is symptomatic, and the probability that the test is accurate, were determined according to the literature.^19^

Of the 1515 facilities, 389 (25·7%) facilities achieved a probability of malaria freedom of at least 95% and sustained this for at least the most recent 36 months based on passive case detection data alone. When combined with data from active case detection, the number of facilities achieving and sustaining a probability of freedom of at least 95% increased to 54·4% (n=824) but ranged from 5·5% (n=83) to 82·6% (n=1251) of facilities by country. Models consistently reproduced patterns in confirmed malaria case data (appendix p 14), and several themes have emerged.

**Figure 2:**
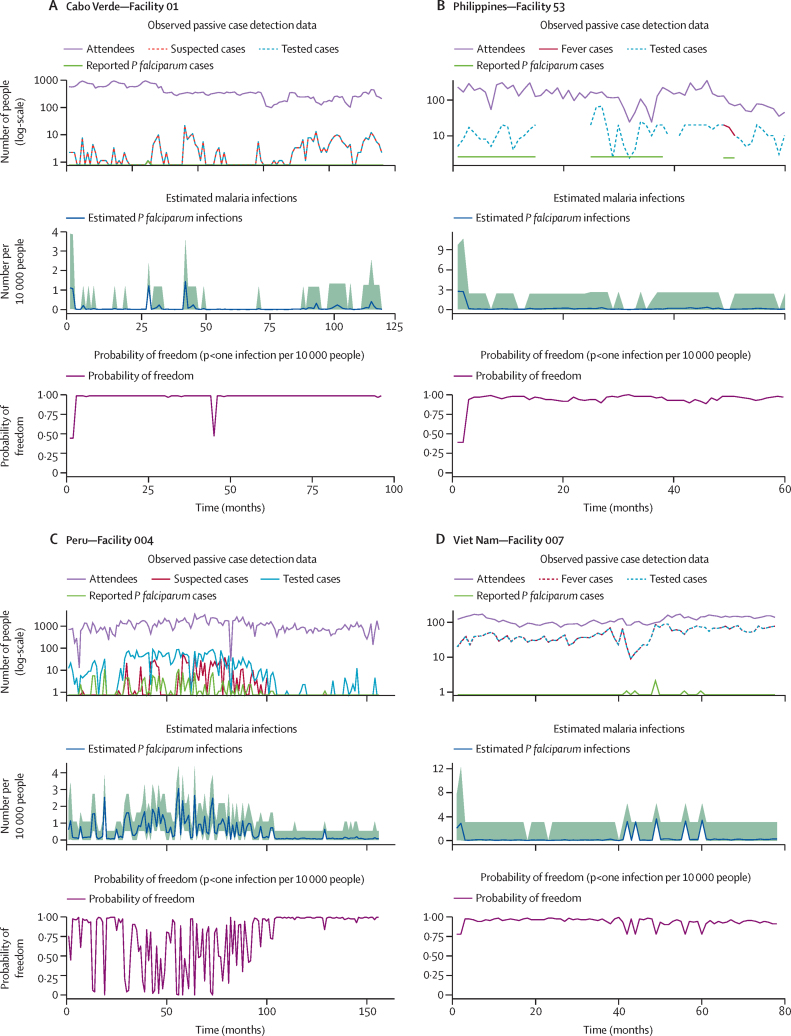
Examples of surveillance system sensitivity results and the corresponding probability of freedom for *Plasmodium falciparum* elimination in selected facilities For each country, the top plot shows the raw data collected at each health facility over the study period. The purple line represents the number of people attending the facility, the red dotted line denotes the number of people with suspected malaria, the blue dotted line shows the number of people tested for malaria, and the green line provides the number of reported *P falciparum* malaria cases reported. The middle plot shows the estimated number of cases in the community that could be present, that the surveillance system should be sufficiently sensitive enough to detect if they exist. This is represented by the blue line: the expected number of cases reflects the ability of the surveillance system to detect infections and is not a prediction of malaria trends in the community. The shaded green area reflects the 95% credible interval around the estimated number of malaria cases per month. The bottom plot for each facility shows the probability of achieving malaria freedom, defined as there being fewer than one case per 10 000 people in the population: when the estimated probability of freedom is 0·95 or higher and zero cases are reported at a health facility, this provides strong evidence of having achieved malaria elimination. The highlighted facilities show that routine malaria surveillance systems alone can achieve and maintain a high probability of freedom if the system capacity and data availability are good.

When considering passive case detection data only to confirm the absence of *P falciparum*, the results suggest that when there are few gaps in routine reporting of malaria data and the health system is strong, routine malaria surveillance is sufficient to achieve a high confidence that malaria elimination has been achieved in the catchment area (figure 2). In these health facilities, for which a high surveillance system sensitivity is achieved, the observed zero malaria cases reported likely accurately reflects the estimated number of malaria cases in the community that should be detected. Therefore, a high estimated surveillance system with zero cases observed translates to achieving and sustaining a high probability of malaria elimination.

There were also several facilities for which the passive case detection data alone was insufficient to achieve a high surveillance system and probability of freedom, despite zero cases being reported. These suboptimal trends were typically driven by either too much missing data, the absence of key input data (eg, the number of attendees or the number tested), not maintaining good testing rates, system factors such as stockouts of supplies or staffing, or a high average travel time. The factors explaining suboptimal surveillance system and probability of freedom varied based on the specific circumstances of each facility.

**Figure 3:**
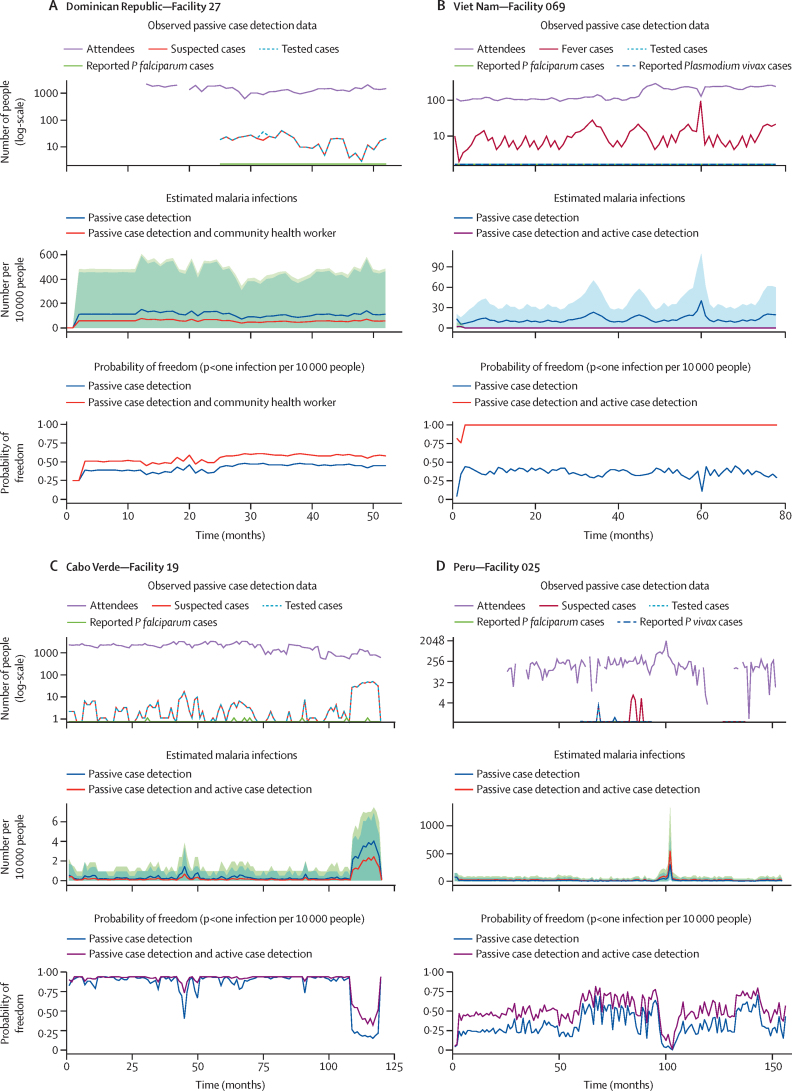
Examples of surveillance system sensitivity results and the corresponding probability of freedom for *Plasmodium falciparum* elimination in selected facilities where active case detection was conducted to supplement information from routine malaria surveillance Results show the different ways to use data collected as part of active case detection to supplement the model and boost the surveillance system and probability of freedom estimates. For each example from a specific facility, the top graph shows the monthly routine malaria surveillance data with the middle showing the estimated surveillance system, and the bottom graph showing the estimated probability of freedom over the observation period. In this case, the second and third plots show the results for two different scenarios: estimates based on the passive case detection data alone and the estimates when the passive case detection and active case detection data are combined. The routine malaria data (top plot) include the number of people attending the facility (purple line), the number of suspected malaria cases (red line), the number of people tested for malaria (blue dotted line), and the number of reported *Plasmodium falciparum* cases (green line). Next, the middle plot shows the results of the estimated number of malaria infections for passive case detection data alone (blue dashed line and blue shaded area depicting the 95% credible interval) and when passive case detection and active case detection data are combined (purple dashed line and greed shaded area for the 95% credible interval). The probability of freedom estimates (bottom plot) are shown for passive case detection data only (blue line) and for passive case detection and active case detection data combined (red line). Different types of active case detection tailored to the epidemiology of each country are shown. (A) Depicts a facility in the Dominican Republic where data from community health workers where populations are screened for malaria monthly improve the overall surveillance system and the probability of freedom. (B) Results are from a health facility in Viet Nam where the active case detection data consist of a household cross-sectional survey using measures of recent (ie, within the previous 12 months) and historical malaria exposure. (C) A facility in Cabo Verde showing the effect of conducting risk-targeted sampling whereby the sample was designed to oversample people who had visited the African continent within the previous 30 days, a population that is 3 times more likely to have malaria exposure compared with non-travellers, strengthening the inference possible in confirming elimination. (D) The example of the health facility in Peru shows the case where the active case detection was designed to target populations less likely to attend the facility given the large distance to seek care, which translated to an overall boost in the probability of freedom by supplementing the available information.

When passive case detection data alone were insufficient to achieve the desired surveillance system sensitivity and probability of malaria freedom, active case detection data were added to supplement the surveillance system sensitivity and probability of malaria freedom estimates. In the Dominican Republic, when the community health worker data were added to that from passive case detection, the uncertainty decreased, leading to an improved surveillance system sensitivity and probability of malaria freedom (figure 3). In Viet Nam, Cabo Verde, and Peru, analysis of the passive case detection data alone showed probability of freedom below the 0·95 threshold in 23 437 (32·2%) of 72 838, 1463 (66·3%) of 2208, and 2273 (3·1%) of 73 944 observations, respectively. However, when including the malaria exposure data collected during the active case detection, the facility-months achieving probability of freedom of 0·95 or higher improved to 35 035 (48·1%), 1746 (79·1%), and 3327 (4·5%) in Viet Nam, Cabo Verde, and Peru, respectively.

**Figure 4:**
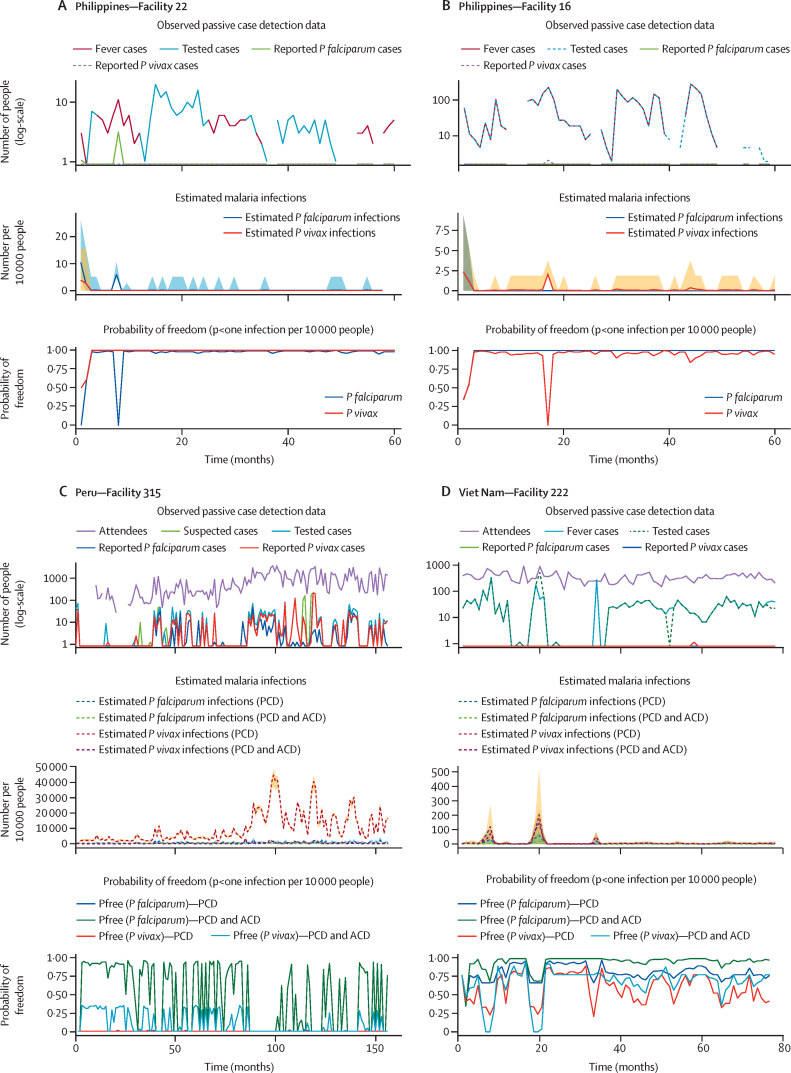
Examples of the Freedom From Infection model results These results show how the model responds when species-specific data are available to generate species-specific estimates. The same series of figures is shown with added lines depicting the *Plasmodium falciparum* and *Plasmodium vivax* specific results. The routine malaria data are shown in the top plot and include the number of fever cases (dark red line), the number people tested for malaria (blue line), the number of reported *P falciparum* cases (green line), and the number of reported *P vivax* cases (purple dotted line). The resulting number of malaria cases potentially present as estimated by the model (middle plot) is shown with the estimated *P falciparum* infections shown with the blue line and the corresponding 95% credible interval with the blue shaded area, whereas the estimated *P vivax* infections are shown with the red line and the corresponding 95% credible interval with the yellow shaded area. The corresponding probability of freedom estimates per species are shown in the final plot with results for *P falciparum* represented by the blue line and *P vivax* by the red line. Examples of two facilities in the Philippines are shown applying the PCD-only model and generating *P falciparum* and *P vivax* specific results for the surveillance system sensitivity and probability of malaria freedom (A and B). These plots show that the results reflect the different data reported, showing the case for which a *P vivax* case was reported which, is reflected in the probability of malaria freedom estimate for that species only and the case for which a *P falciparum* case was reported with no impact on the *P vivax* estimates. Similarly, facilities in Peru (C) and Viet Nam (D) show the case where species-specific information on recent exposure within the past 12 months and historical exposure to each species collected during ACD activities were available for both species. As shown in both examples, when the species-specific ACD data are combined with that from PCD (green dotted lines for *P falciparum* and purple dotted lines for *P vivax*) the estimates of surveillance system sensitivity become more precise and the probability of malaria freedom increases compared with the case if only PCD data were available (blue dotted lines for *P falciparum* and red dotted lines for *P vivax*). ACD=active case detection. PCD=passive case detection.

Applying the passive case detection only model for which *P falciparum* and *P vivax* data were available, species-specific estimates of both surveillance system sensitivity and probability of malaria freedom were generated: when a *P falciparum* or *P vivax* case is reported the corresponding surveillance system and probability of freedom estimates react accordingly (figure 4). The species-specific model was also applied when data from both passive case detection and active case detection were available. The results from facilities in both Peru and Viet Nam show similar trends where both the estimates of surveillance system sensitivity and probability of malaria freedom as well as the corresponding active case detection-associated boost follow the expected species-specific trends (figure 4).

**Figure 5:**
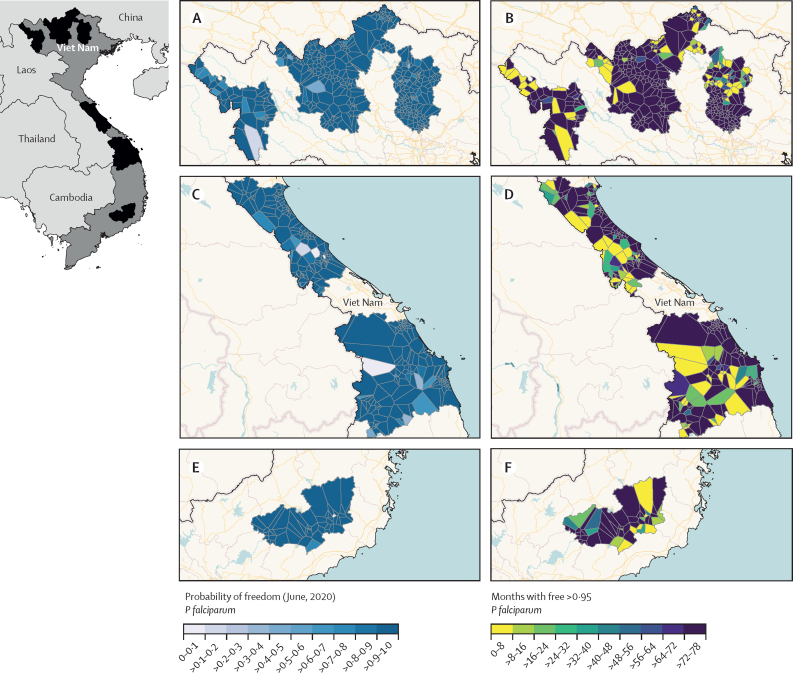
Probability of freedom from *Plasmodium falciparum* in Viet Nam Results are shown for each of the catchment areas of the 921 facilities included in the three study regions in Viet Nam: Northern (A and B), Central (C and D), and the Central Highlands (E and F), with the location of each study region within the country shown in the inset map to the left of the panel. The figures on the left (A, C, and E) show the probability of malaria freedom estimate per catchment area at the final month of observation with the darker the blue showing the higher the estimated probability of freedom. The series of maps on the right (B, D, and F) show how many months from the final timepoint that each facility was able to maintain a probability of malaria freedom greater than 0·95, with the facilities in the teal to blue end of the spectrum meeting the WHO criteria for maintaining zero cases and a strong surveillance system for at least 36 months. Facilities with a probability of malaria freedom <0·95 represent those where there is high uncertainty in the data available and those with <0·50 meaning transmission and elimination are equally likely outcomes.

In Viet Nam, data were collected from 921 health facilities across three regions of the country. The results for *P falciparum* suggest that, at the end of the study period, most facilities had achieved a high estimated probability of malaria freedom (figure 5). However, only 738 (80·1%) of 921 facilities had sustained a probability of malaria freedom of 0*·*95 or higher for the last 36 months of the study period. The results for *P vivax* showed patterns consistent with those of *P falciparum* with 722 facilities sustaining probability of malaria freedom for at least 3 years (not shown).

## Discussion

We applied the FFI framework in five diverse malaria transmission settings and surveillance landscapes, finding that, despite the intercountry heterogeneity, several themes emerged, supporting the FFI framework as a programmatic tool for malaria elimination. First, the FFI framework identified factors associated with a strong passive malaria surveillance system and, conversely, those factors that could be improved to strengthen the system; these are consistent with existing knowledge, which suggests that the framework has generated credible results.^20^, ^21^, ^22^, ^23^ Notably, we confirm that high-performing routine surveillance systems alone were sufficient to achieve a high degree of confidence that malaria elimination has been achieved. Additionally, we show that there are options to boost confidence in elimination, including community case management and active case detection, to supplement probability of malaria freedom estimates when passive case detection data alone is insufficient. Finally, the species-specific extension of the framework was successfully applied in multiple settings where *P falciparum* and *P vivax* are co-endemic. The resulting estimates of species-specific surveillance system sensitivity and probability of malaria freedom provide national malaria programmes with the opportunity to monitor progress in a way that can be specific to the type of malaria targeted for elimination.

The FFI framework was designed to support data-driven decision making for strengthening malaria surveillance and to provide evidence to support the confirmation of elimination. Ideally, this tool would be integrated into an existing platform or web-based application to ensure user friendliness as part of an iterative information cycle. For example, if all facilities come back with a strong surveillance system, the corresponding action would be to maintain the status quo. In contrast, if the results suggest a need for improving the system, national malaria programmes could use the results to identify which specific health facilities to improve and how. Potential reasons why a facility might be underperforming can range from facility-specific to broader trends; addressing them requires a holistic approach whereby both the sensitivity table as well as facility-specific outputs are examined to tease apart potential drivers and logical next steps. Depending on the goals of the programme, the results of the FFI model could be used to inform steps to improve performance, whether it be improving access to care, increasing testing, or reducing missing data. Alternatively, the results could inform whether additional surveillance components to supplement the information obtained through passive case detection are warranted. For example, if access to care is a consistent challenge, then national malaria programmes could consider incorporating community case management. Once the system is strong, national malaria programmes could then use this tool to track progress from the health facility to the regional level, supporting claims of subnational verification of elimination.

A strong and effective surveillance system is one in which input data are valid and the surveillance system is both efficient and effective at capturing the trends it was designed to measure. The FFI framework applied here is one of many tools that can assess the effectiveness of surveillance systems. Although a comprehensive review of all available tools is beyond the scope of this discussion, this type of review typically falls into two domains: data quality assessments and more comprehensive reviews of the surveillance system.^24^, ^25^ Data quality assessments typically review facets of system efficiency including timeliness, completeness, accuracy, and consistency with the aim of assessing both data quality and the overall data management system. Key indicators of data completion and timeliness are routinely monitored, but depend on reporting frequency; more in-depth data quality assessments are labour-intensive and as such are typically limited to a small number of facilities, limiting their generalisability. Several tools also exist that support a more in-depth review of the system including data collection, integration, and use in decision making (eg, malaria surveillance toolkits and health information system stages of continuous improvement). The broader system review tools provide a standardised framework and indicators focused on evaluating system performance, infrastructure, processes and collection, and the use of data.^24^ The toolkits have been designed in a modular format so countries can adapt the scope and content to their setting. Data are collected on each selected domain and include document reviews, data quality assessment, and key informant interviews. However, in addition to being labour-intensive to complete, the toolkits use a qualitative or descriptive approach to synthesise and interpret the collected data and rely on the more subjective expert opinion.

Although the FFI framework has some important limitations, it provides important advantages and synergies within the existing global landscape of malaria surveillance. Firstly, the FFI tool approaches the issue of surveillance improvement from a dynamic modelling perspective, largely relying on routinely collected data and redoubling the inferential value. Such an approach means that information on multiple facilities across the country can be assessed as frequently as data become available. Secondly, the framework uses a data-driven, Bayesian approach that generates the associated degree of uncertainty around estimates and thus informs on the strength of inferences possible. Finally, one key area of evaluating malaria surveillance systems that the existing tools do not address is the ability of the system to detect malaria in the community (ie, the overall sensitivity of the surveillance system). Understanding the degree of malaria burden not detected by the surveillance system is a known problem and a function of the degree of the asymptomatic malaria reservoir.^26^ In elimination settings, the ability of surveillance systems to detect infections in the community is particularly salient given that claims of elimination rely on proving a zero.^5^ The FFI framework was developed to explicitly address this issue by measuring the probability that, if any infections were present, the surveillance system in place, either through passive case detection alone or a combination of passive case detection and active case detection, would detect them.^6^ Although facilities can never reach a probability of freedom of 100% (ie, there are no undetected infections in the community), by applying the framework and achieving and sustaining a high probability of freedom, we can be confident that, if there was any sustained transmission, it would lead to a symptomatic case and thus be detected. Estimating the sensitivity of the malaria surveillance system is an important advantage of the FFI framework that is not addressed with existing tools. Despite using different approaches to data collection and analysis, the FFI framework offers important synergies with existing tools that should be further explored.

Notably the definition of elimination applied within the context of the FFI framework is different from that used by WHO as part of the elimination certification process in important ways.^5^ Firstly, within the FFI model, elimination is defined as achieving and sustaining a probability of freedom greater than 0·95. The probability of freedom metric, derived using the surveillance system sensitivity parameters, is therefore akin to a negative predictive value of the surveillance system, or the probability that a negative report from the surveillance system likely reflects zero infections in the population. In contrast, WHO considers malaria elimination as achieved if there are 3 consecutive years with zero locally acquired infections and having a surveillance system in place that is sufficient to prevent reintroduction. The focus on a strong surveillance system is consistent, with the WHO definition applying a qualitative review of evidence. In contrast, the FFI framework uses a quantitative approach to make this assertion. Although the two approaches to defining elimination are expected to be complimentary, a more in-depth comparison of the methods and any implications for subsequent conclusions is required.

Although applying the FFI framework provides detailed and granular information, broad application of the FFI framework requires development of a tool that can be integrated into existing health information systems that provides the corresponding granularity of input data required to inform estimates. Here, we analysed data per facility at a monthly timescale and relied on interviews with health facility staff to obtain information on health systems parameters. The data required by this analysis is routinely collected within the health system but is typically stored within different databases (eg, malaria registries, commodities tracking, and training activities) or digitised at an aggregated spatial scale (eg, district). Similarly, this model also relies on an estimate of the size of the health facility catchment population, which is not routinely available.^27^ Digital data collection for malaria surveillance is being scaled up in many countries and would facilitate routine application. Extending the FFI framework to adopt data from additional surveillance components including foci investigation, entomological surveillance, and the case of imported or relapse infections could be important components for future development.

As part of this study, serological markers of recent and historical exposure were measured in the active case detection components. When confirming the absence of transmission in a population, maintaining strong evidence over time is crucial to ensuring confidence that the goal of elimination has been achieved. Therefore, the timestamp associated with the information garnered from the different diagnostics, including the increased detection window of residual histidine-rich protein II when interpreting rapid diagnostic test results, is just as important as the overall sensitivity or limit of detection of the test.^28^ For example, although PCR is highly sensitive, that data becomes much less relevant over time as any infections outside of that detection window are not captured. In contrast, serological measures of exposure provide evidence of absence of transmission over a much longer period, with the specific window dependent on the kinetics of the antigens used. Although the sensitivity of detecting an infection is higher than for metrics of exposure,^18^ that the information available represents exposure over a much longer time frame translates to a larger increase in the overall sensitivity of the surveillance system and probability of freedom.^29^

There are limitations to note. First, the variables used to estimate the probability of seeking care and the probability that a person is tested for malaria—the key input parameters to quantify the surveillance system sensitivity and the corresponding probability of malaria freedom—could be proxies for underlying trends. However, given that some of the processes involved in the care-seeking pathway that must be represented in the FFI framework are qualitative in nature, such proxy variables might be sufficient. Additional validation of the inputs to estimate the surveillance system sensitivity, identifying the minimal set required to generate appropriate estimates and any variation between settings, would facilitate data collection, interpretation of the results, and use of the outputs to guide targeted interventions. Second, there is an implicit model assumption that routine malaria surveillance data have already passed existing data quality checks. No additional built-in data quality or validity checks are included in the model beyond missing data corresponding to increased uncertainty in estimates.^6^ Third, the health systems parameter data were largely collected using key informant interviews at a single timepoint and were applied uniformly over the observation period. Ideally, these data could be collected continuously and thus modelled as a time varying variable; however, generating these data was not possible within the scope and timeframe of this study. Fourth, the species-specific model focused solely on *P falciparum* and *P vivax* and did not include extensions for *Plasmodium malariae* or *Plasmodium ovale.* This was due to these species not being reported as part of passive case detection in the included health facilities. Incorporating all species of human malaria into the model framework is an important next step. Finally, the FFI framework does not include a full transmission model. However, if transmission were present, a fraction of infections would present for care and be identified through passive case detection. Therefore, for the context of FFI, our approach is reasonable.^26^

The FFI framework provides an approach to quantify and holistically interpret routine malaria data from health facilities that compliments currently used frameworks.^24^, ^30^ The FFI model is ideally integrated into decision making as part of an iterative process whereby results are continuously updated to track and maintain progress over time. Working as part of a feedback loop becomes particularly salient if interventions are applied to improve the system: the FFI results could inform which improvements would be most beneficial and monitor any subsequent change. Ultimately, confirming the absence of any malaria infections is only possible if the entirety of the population is sampled with a perfect diagnostic test. Given that this is practically impossible, the FFI model could provide a needed framework to determine the likelihood that malaria elimination has been achieved.

### Contributors

### Data sharing

The data used as part of this work consists of both data from the national malaria programmes in the respective setting as well as prospectively collected data for research purposes. All data included in this work are available upon reasonable request by contacting the corresponding author.

## Declaration of interests
