# Supplementary material for: Evidence-based decision making for malaria elimination applying the Freedom From Infection statistical framework in five malaria eliminating countries: an observational study
Source: Lancet Glob Health. 2025 Aug 19;13(9):e1591–604. doi: 10.1016/S2214-109X(25)00236-0 (PMC12368415; doi:10.1016/S2214-109X(25)00236-0)
Supplement: Supplementary appendix [file mmc1.pdf]

THE LANCET
Global Health

Supplementary appendix

This appendix formed part of the original submission and has been peer reviewed. We post it as supplied by the authors.

Supplement to: Stresman G, Nelli L, Wu L, et al. Evidence-based decision making for malaria elimination applying the Freedom From Infection statistical framework in five malaria eliminating countries: an observational study. *Lancet Glob Health* 2025; **13:** e1591–604.

Appendix - Evidence-based decision-making for malaria elimination: An observational study applying the Freedom From Infection statistical framework in five malaria eliminating countries

Table of Contents

SUPPLEMENTARY TABLE 1 - OVERVIEW OF STUDY AREAS AND DATA COLLECTION ACTIVITIES IN EACH OF THE FIVE STUDY COUNTRIES WHERE THE FREEDOM FROM INFECTION FRAMEWORK WAS APPLIED. 2

SUPPLEMENTARY FILE 1 – GUIDELINES FOR DATA NEEDS TO INFORM THE FREEDOM FROM INFECTION MODEL . 5

SUPPLEMENTARY TABLE 2 – MALARIA ANTIGENS USED TO CLASSIFY RECENT AND HISTORICAL *P. FALCIPARUM* AND *P. VIVAX* EXPOSURE. 7

SUPPLEMENTARY FILE 2 – DETAILED STATISTICAL METHODOLOGY FOR FFI MODEL EXTENSIONS................... 8

SUPPLEMENTARY TABLE 3 – RESULTS OF METRICS OF MALARIA INFECTION AND EXPOSURE FOR BOTH *P. FALCIPARUM* AND *P. VIVAX* FROM THE CROSS-SECTIONAL 'FREEDOM' SURVEYS IN CABO VERDE, PERU, AND VIETNAM 13

SUPPLEMENTARY FILE 3: POSTERIOR PREDICTIVE CHECKS - POSTERIOR PREDICTIVE CHECKS FOR MODEL FIT (CABO VERDE CASE STUDY).. 14

Supplementary Table 1 - Overview of study areas and data collection activities in each of the five study countries where the Freedom From Infection framework was applied.

	Cabo Verde	Dominican Republic	Peru	Philippines	Vietnam
WHO Region	African	Americas	Americas	Western Pacific	Western Pacific
**Malaria**					
Study Area	National	Santo Domingo and Districts bordering Haiti	53 Districts in the Loreto Department	TayTay, Roxas, Bataan Municipalities	Bac Can, Dien Bien, Ha Giang, Kon Tum, Lam Dong, Lao Cai, Quang Binh, Quang Nam, Quang Ngai, Quang Tri, Thai Nguyen, Yen Bai Provinces
Epidemiology	-Archipelago with 9 inhabited islands off coast of Senegal<br>-Last locally acquired case in January 2018	- Island of Hispaniola, which is the last malaria endemic island in the Caribbean and is shared with Haiti<br>-Pre-elimination status with low burden and a small number of active foci	- Cases numbers decreasing with implementation of intensified control strategies<br>- Includes several remote communities >5 km from the nearest health center	- Northern Palawan had zero reported malaria cases but risk of reintroduction from the active foci in south Palawan and frequent travelers visiting the area. Moron was declared malaria free in 2018 with the	-Diverse transmission across the different regions: Northern area has been low/pre-elimination for several years whereas Central and Southern regions have only recently achieved pre-elimination status

				last indigenous case reported in 2011.	
Primary Anopheles vectors	*An. arabiensis*	*An. albimanus*	*An. darlingi*	*An. flavirostris*	*An. minimus s.l.*; *An. sinensis*; *An. aconitus*; *An. vagus*
Plasmodium species investigated	*P. falciparum*	*P. falciparum*	*P. falciparum*; *P. vivax*	*P. falciparum*; *P. vivax*	*P. falciparum*; *P. vivax*
FFI Study Objective	Risk-targeted sampling	Community health workers (CHWs)	Populations less represented by routine malaria surveillance	Multiple species of malaria parasites	Large spatial scale
**Routine malaria surveillance**					
N Health Facilities	26	34	474	57	921
Time period of routine monthly data	2014 to 2021	2019 to 2022	2010 to 2022	2014 to 2020	2014 to 2020
Diagnostic test	RDT (SD Bioline)	RDT, microscopy	microscopy	RDT (Abott Bioline Pf/Pv), microscopy	RDT (SD Bioline Pf/Pv), microscopy
First line antimalarial drug *(P. falciparum / P. vivax)*	Artemisinin Lumefantrine + low dose Primaquine / NA	Chloroquine / NA	Artesunate-Mefloquine + high-dose Primaquine / Chloroquine + Primaquine	Artemisinin Lumefantrine + low dose Primaquine / Artemisinin Lumefantrine + Primaquine	Dihydroartemisinin Piperaquine + low dose Primaquine/ chloroquine + Primaquine
**Active Surveillance**					
Study Area	Praia (Santiago Island), Sal Rei	Santo Domingo	Belen, Indiana	Morong, Bataan	Bac Mae, Bo Trach

	(Boavista Island)				
Sampling Design	Reactive respondent driven sampling targeting recent travellers to the African continent	All community health workers working within selected facilities	Stratified selection of villages according to distance to facilities as a proxy for access	Retrospective analysis on samples from Bataan.	Random selection of households residing in catchment areas of all facilities in district and 2-stage random sample of facilities and households within catchment areas of selected facilities
N Health Facilities	26	9	16	1	28
N people sampled	725	12 CHWs	4000	2059	3983
Diagnostic test	RDT, pooled PCR, Luminex	RDT	microscopy, pooled PCR, Luminex	Pooled PCR, Luminex	RDT, microscopy, RT-PCR, Luminex
Abbreviations: RDT = rapid diagnostic test; PCR = polymerase chain reaction; RT-PCR = real-time polymerase chain reaction; Luminex = platform used for multiplex antibody testing.					

Supplementary File 1 – Guidelines for data needs to inform the Freedom From Infection model

Applying the Freedom From Infection (FFI) model has some specific data needs to inform estimates of both SSe and Pfree. Here, we provide a summary of the requirements to facilitate implementation of these tools where programs may be interested in applying them. Overall, all data whether from routine passive case detection (PCD) or any form of active case detection (ACD) need to be anchored to a health facility and a month. In other words, the spatial unit of analysis is the health facility, and requires the *spatial coordinates of the facility* as well as the *size of the catchment population* being represented (however loosely defined), and the temporal unit of analysis is a month. The specific data requirements per type of data are presented below and should be adapted as needed based on the context of the specific surveillance system.

<u>Routine PCD Data</u>

Per facility, per month:

- Number of people attending the facility
- Number of people suspected of having malaria
    - Number of fever cases can be used as a proxy for suspected, but results impacted by the degree of non-malarial fevers in the population
- Number of people tested for malaria
- Number of people testing positive for malaria by diagnostic test type and species
- Number of malaria cases confirmed to be imported

<u>Malaria Surveillance System Data</u>

Per facility, per month is ideal OR data on the month and location when these events occurred. The key data that were associated with factors in the care-seeking cascade in the tested case studies included:

- Availability of malaria drugs
- Availability of malaria tests
- Training in malaria testing, case management
- Training on and regular reporting of malaria surveillance data

- Availability of guidelines for malaria testing and case management
- When supervisory or competency assessment visits on case management or testing occurred

Note that other information may be relevant to how a specific malaria surveillance system operates and should be tailored accordingly. For example, the number of physicians or clinical officers per capita, or a metric of how well malaria guidelines are applied could be relevant in given settings.

<u>Active Case Detection</u>

Active Case Detection (ACD) data can take multiple forms. The most common may be household surveys where people are tested for malaria but can also consist of community case management (CCM) activities where workers visit households to test for malaria. Overall, all data must be connected to a health facility and month. The minimum dataset to incorporate any data from ACD consist of:

- Number of people tested for malaria
- Which diagnostic tests were used and the corresponding test sensitivity and specificity for each
- Number of malaria positive individuals, which diagnostic test was used, which malaria species
- Imported or locally acquired status for any malaria infections detected

Note that more complex analytic approaches are possible if the data are available to support the inference. For example, age structure of the population sampled, information on any known individual- or population-level risk factors for malaria or environmental factors.

Supplementary Table 2 – Malaria antigens used to classify recent and historical *P. falciparum* and *P. vivax* exposure.

**Species**	**Antigen**	**Description**	**Exposure period**	**Time (Years)**
P. falciparum	PfAMA1	Apical membrane antigen 1	Historic	5
P. falciparum	PfMSP1_19	Merozoite surface protein 1-19	Historic	5
P. falciparum	Etramp 5 Ag 1	Early transcribed membrane protein 5 antigen (exon) 1	Recent	1
*P. vivax**	PvMSP119	Merozoite surface protein 1-19	Historic	5
*P. vivax**	PvAMA1	Apical membrane antigen 1	Historic	5
*P. vivax**	PvRBP2b	*P. vivax* erythrocyte binding protein	Recent	1
*Samples from Cabo Verde were not tested against P. vivax antigens because this specie was not endemic.				

Supplementary File 2 – Detailed statistical methodology for FFI model extensions

The data collected from both the routine surveillance system and health system interviews was collated and used to inform the foundation of the Freedom From Infection (FFI) modelling framework as described in Nelli et al.[7] Briefly, the FFI analytical framework was used to estimate the probability that an individual would seek care, the probability of a clinician suspecting malaria (where this data was collected), and the probability of being tested for malaria. The other components in the care-seeking cascade used to estimate malaria SSe (e.g., probability of being infected, probability of being symptomatic, and probability that the test is correct if infected) are not measurable parameters and were estimated according to the published literature. The next step in the modelling approach was to use the estimated probabilities and overall cascade to estimate the SSe, here defined as the difference between the observed and expected number of cases in the community that should be detected by the surveillance system. Where there is a gap between these two model outputs, this would be interpreted as that facility having a low SSe. When they overlap, the facility has a strong sensitivity at detecting malaria infections if they exist above the pre-defined threshold of 1 case per 10,000 people. The estimated SSe is then used to determine the probability that the area is free from malaria infection according to the defined threshold (Pfree). Therefore, if the estimated Pfree is high, and no cases are reported, we can be confident that there are fewer than 1 case per 10,000 people in the population, otherwise the system would have detected them. The results of the FFI models were plotted over time and mapped to assess temporal and spatial trends, respectively.

Given that PCD is the cornerstone of all malaria surveillance systems, the initial model framework was developed using this foundation. However, one of the main advantages of the FFI approach is the ability to combine different sources of surveillance information, where available, to maximize the information with which to inform decision-making for strengthening

the surveillance system and informing elimination. In this respect, as part of this project we expanded the model framework to explore the added value of integrating i) Community Health Workers data, ii) PCD in the health facility with multiple infection diagnostic tests (RDT, microscopy), iii) multiple *Plasmodium* species in both PCD and ACD and iv) ACD with measures of infection (RDT, microscopy, PCR) and seroprevalence data on both recent and historical exposure to malaria.

i) Integration with Community Health Workers data

Although none of the communities selected as part of the FFI case studies had community case management in place, this is a common intervention applied in many malaria endemic settings and is typically implemented as part of foci investigation in elimination settings. Thus, this was an important model extension to develop to support future and broader application of the framework. To estimate the added value of community case management, we included further steps when modelling the observation process, using data that would typically be collected by community health workers (CHW). Like eq. (8), and we modelled the number patients attending a community health worker (A_{CHW}) as

$$A_{CHW_{i,j}} \sim Pois\left(\lambda_{\alpha_{i,j}}\right) \tag{17}$$

Similar to eq. 12, we modelled the number of patients tested for malaria (T_{CHW}), as:

$$T_{CHW_{i,j}} \sim Pois\left(P_{TEST_{CHW_j}} \lambda_{FEV_{i,j}}\right) \tag{18}$$

with $logit(P_{TEST_{CHW_j}})$ being a simple function of a different intercept for each CHW.

Finally, similar to eq. (13) and (14), we modelled the number of patients confirmed with malaria as

$$C_{CHW_{i,j}} \sim Pois\left(\lambda_{CONF_{CHW_{i,j}}}\right) \tag{19}$$

with the rate $\lambda_{CONF_{CHW_{i,j}}}$ defined as

$$\lambda_{CONF_{CHW_{i,j}}} = P_{CLINICAL} P_{TEST_{CHW_j}} M_{i,j} \tag{20}$$

Note that all these steps of the CHW-related observation process, share the same estimation of the underlying malaria transmission ($M_{i,j}$). In this way, in the health facilities where CHW were available, in addition to standard monitoring, we could obtain two different estimates of $M_{i,j}$ and $Pfree_{i,j}$ (one with HF data alone, one with HF+CHW data).

ii) Integration with multiple infection diagnostic tests

In case PCD are collected using both Microscopy and RDT, some steps of the observation process can be split in two. For example, if the number of people tested with microscopy $T_{Mic_{i,j}}$ is different from the number of people tested with RDT $T_{RDT_{i,j}}$, we can model them with two different processes, and eq. (12) would be replaced by

$$T_{Mic_{i,j}} \sim Pois\left(P_{TEST_{Mic_j}} \lambda_{FEV_{i,j}}\right) \tag{21}$$

and

$$T_{RDT_{i,j}} \sim Pois\left(P_{TEST_{RDT_j}} \lambda_{FEV_{i,j}}\right) \tag{22}$$

Where $P_{TEST_{Mic_j}}$ and $P_{TEST_{RDT_j}}$ can be modelled as in eq. (16), each with their own set of covariates.

When considering the confirmed cases, we can split eq. (13) and (14), into two different processes, one for microscopy-confirmed and one for RDT-confirmed cases (respectively $C_{Mic_{i,j}}$ and $C_{RDT_{i,j}}$), as

$$C_{Mic_{i,j}} \sim Pois\left(\lambda_{CONF_{Mic_{i,j}}}\right) \tag{23}$$

with the rate $\lambda_{CONF_{RDT_{i,j}}}$ defined as

$$\lambda_{CONF_{Mic_{i,j}}} = P_{CLINICAL} P_{TEST_{Mic_j}} M_{i,j} \tag{24}$$

and

$$C_{RDT_{i,j}} \sim Pois\left(\lambda_{CONF_{RDT_{i,j}}}\right) \tag{25}$$

with the rate $\lambda_{CONF_{RDT_{i,j}}}$ defined as

$$\lambda_{CONF_{RDT_{i,j}}} = P_{CLINICAL} P_{TEST_{RDT_j}} M_{i,j} \tag{26}$$

As well as in the previous special case (integration with CHW data), again all these steps of the diagnostic test-related observation process, share the same estimation of the underlying malaria transmission ($M_{i,j}$). In this way, when data from one diagnostic test is patchy, the model can make inference from the combination of the two sets, and we can obtain a more robust estimation of $M_{i,j}$ and $Pfree_{i,j}$.

iii) Multiple *Plasmodia* species

The models presented so far, only describe cases as "malaria", but not specific for either *P. falciparum* or *P. vivax* which present different challenges for elimination and surveillance. We further modified the state process (eq. from (1) to (7)) and each step has been split in two. For example, we can split eq. (1) and (2) into

$$M_{Pf_{i,j}} = E_{Pf_{i,j}} + O_{Pf_{i,j}} \tag{27}$$

$$E_{Pf_{i,j}} \sim Pois(\lambda_{E_{Pf_{i,j}}} N_{i,j}) \tag{18}$$

for *P. falciparum*, and

$$M_{Pv_{i,j}} = E_{Pv_{i,j}} + O_{Pv_{i,j}} \tag{27}$$

$$E_{Pv_{i,j}} \sim Pois(\lambda_{E_{Pv_{i,j}}} N_{i,j}) \tag{28}$$

for *P. vivax*, and so on for the rest of the state process.

Similarly, in the observation process, we can split the reported cases into *Plasmodium*-specific reported cases, reconstruct the *Plasmodium*-specific underlying malaria transmission and the *Plasmodium*-specific Pfree.

Note that in most case studies where *P. vivax* is/was endemic, a combination of both multiple diagnostic test and multiple *Plasmodia* species was available (e.g., Peru, Philippines, Vietnam).

iv) Integration with ACD using infection and seroprevalence data.

In the state process, $\lambda_{E_{i,j}}$ is the force of infection related to the proportion of individuals in the population who are expected to have malaria at any given time. If seroprevalence data is available in the catchment population j^{th} facility, $\lambda_{E_{i,j}}$ can be shared between the different sources of data (PCD and seroprevalence), and it can be simultaneously estimated by two different processes.

We did this, by adding a likelihood in the model, hence estimating the same $\lambda_{E_{i,j}}$ generating the same latent malaria cases, but using serology data, as:

$$S^{+}{}_{j} \sim Bin(P_j, (1 - \pi_j)) \tag{29}$$

where $S^{+}{}_{j}$ is the total positive cases for a given marker in the catchment population of the jth facility, P_j is the population tested for seroprevalence, and $(1 - \pi_j)$ is the probability that

probability that a person was not exposed to malaria in the window of validity of that marker. The estimation of π_j depends on the marker used, and the assumed temporal validity. Hence, we calculated it as

$$\pi_j = \prod_{k=1}^{v} (1 - \lambda_{E_{(t-k),j}}) \quad (30)$$

where t is the month in which the serology samples have been collected, and v is the window of vailidity for that specific marker. Note that this can be split for different malaria species, hence informing the different forces of infection (e.g., $\lambda_{E_{Pf_{i,j}}}$ and $\lambda_{E_{Pv_{i,j}}}$).

Also note that different markers can be included in the analysis, for example we can use markers for recent exposure, with, say, $v = 12\ months$ and markers for historical exposure with $v = 240\ months$. In such case we can calculate different π_j for different markers, but both informing the same λ_E.

When infection data (i.e., PCR, RDT, microscopy) is available at a given time t is available, prevalence data can also provide a "boost" proportional to the sensitivity and specificity of the test used, by contributing to the estimation of λ by

$$A^{+}_{t,j} \sim Bin(A_{t,j}, \omega\lambda_{E_{t,j}}) \quad (31)$$

where $A^{+}_{t,j}$ is the number of positive cases in the catchment population of the jth facility at time t over the total number of people sampled. The model extension integrating the infection data has not been extensively tested to date as efforts were concentrated on the more technically difficult piece of integrating the serological data.

As in the previous special cases (integration with CHW and multiple species), all these added steps share the same estimation of force of infection, hence the underlying reconstructed malaria transmission ($M_{i,j}$). In this way, although seroprevalence is not time specific (i.e., it only tells if an individual was exposed at some point in the past v months), its integration with the PCD data in the same catchment population, corroborates the evidence that we get from the PCD data alone, hence allowing to get a "boost" in the estimation of Pfree.

Supplementary Table 3 – Results of metrics of malaria infection and exposure for both *P. falciparum* and *P. vivax* from the cross-sectional 'freedom' surveys in Cabo Verde, Peru, and Vietnam

	Cabo Verde	Peru		Vietnam	
	P. falciparum	*P. falciparum*	*P. vivax*	*P. falciparum*	*P. vivax*
N samples	725	4000		3982	
RDT positive – n (%)	3 (0)	0 (0)	0 (0)	2 (0)	1 (0)
Microscopy positive – n (%)	–	0 (0)	0 (0)	0 (0)	0 (0)
PCR positive – n (%)	0 (0)	0 (0)	0 (0)	0 (0)	0 (0)
Recent Exposure – n (%)	8 (1.1)	19 (0.5)	186 (4.7)	1 (0)	26 (0.7)
Historical Exposure – n (%)	33 (4.6)	82 (2.1)	217 (5.4)	46 (1.1)	115 (2.9)

Supplementary File 3: Posterior Predictive Checks - Posterior Predictive Checks for Model Fit (Cabo Verde Case Study)

To assess model adequacy, we conducted posterior predictive checks (PPCs) for each of the five country case studies. These checks are well suited to our framework, which aims to reconstruct latent malaria infections under imperfect surveillance. Because the model explicitly accounts for under-ascertainment, observed case counts are not expected to match the predicted latent burden directly. The goal of the PPCs is therefore not to verify predictive accuracy in the usual sense, but rather to assess whether observed data are plausible realisations from the fitted model, given its structure and uncertainty.

We focused the PPCs on the number of confirmed malaria cases reported at each health facility over time. For each facility-month, replicated datasets were generated by simulating from the model's likelihood using posterior samples. From these, we computed posterior means and 95% credible intervals, and compared them with the observed values.

Figure SX1 presents the PPC time series for Cabo Verde (P. falciparum only) as a representative example.

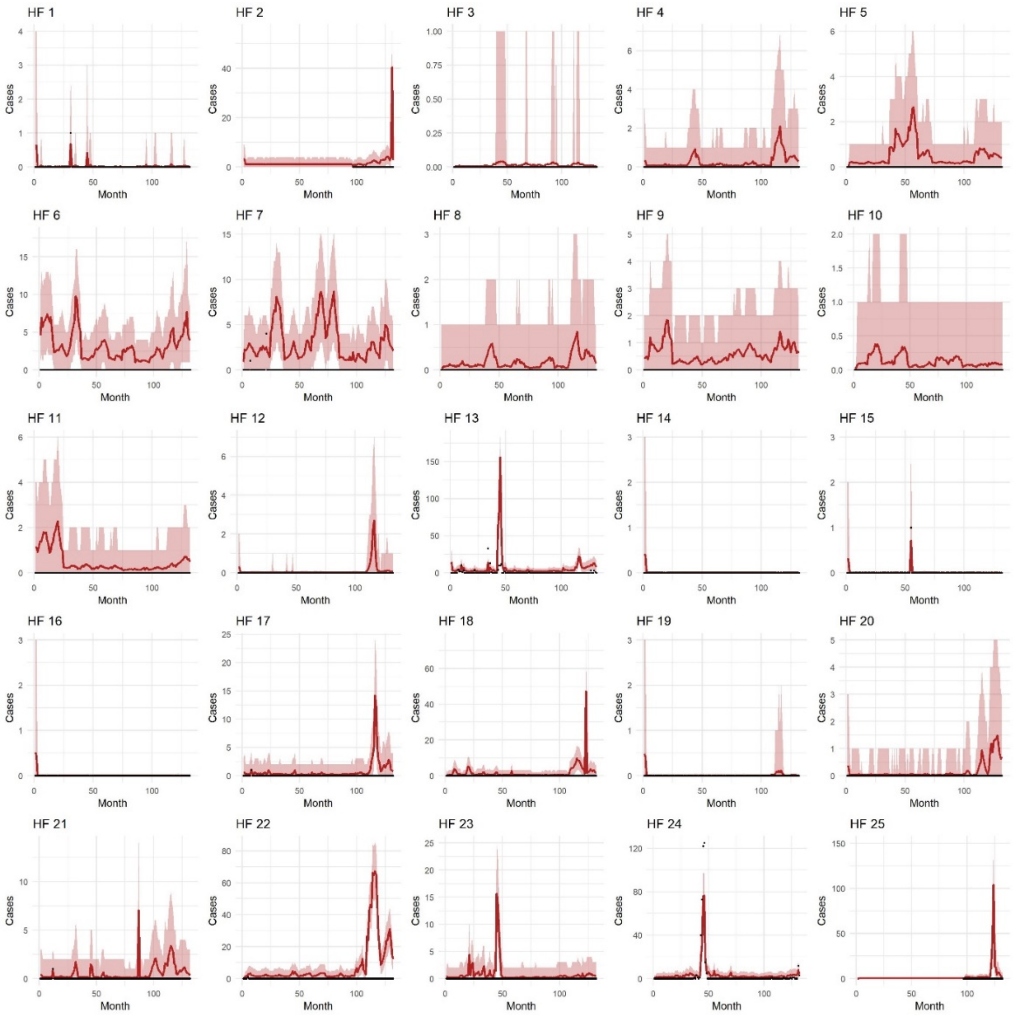

Figure SX1. Posterior predictive checks for confirmed malaria cases at each health facility in Cabo Verde. Each panel shows the monthly number of confirmed malaria cases reported at a specific health facility (black points), compared against the model's posterior predictive distribution. The solid red line indicates the posterior mean prediction, and the shaded red band represents the 95% credible interval.

Summary of Predictive Coverage

Table SX1 reports the **percentage of observed confirmed case counts** falling within the 95% credible intervals of the model's posterior predictive distribution for each country and species.

Table SX1. Posterior predictive coverage of confirmed malaria case counts by country and Plasmodium species. Values indicate the percentage of observed monthly confirmed case counts that fall within the 95% credible intervals of the model's posterior predictive distributions. Results are stratified by *P. falciparum* and *P. vivax* where applicable.

Country	*P. falciparum* (%)	*P. vivax* (%)
Cabo Verde	94.6	-
Dominican Republic	85.3	-
Peru	96.2	83.0
Philippines	97.6	97.3
Vietnam	91.1	87.7

These results show good agreement between observed and predicted distributions across diverse transmission settings and surveillance capacities, providing reassurance that the model is consistent with empirical case data despite its latent structure.
